# Design and evaluation of the computer-based training program Calcularis for enhancing numerical cognition

**DOI:** 10.3389/fpsyg.2013.00489

**Published:** 2013-08-05

**Authors:** Tanja Käser, Gian-Marco Baschera, Juliane Kohn, Karin Kucian, Verena Richtmann, Ursina Grond, Markus Gross, Michael von Aster

**Affiliations:** ^1^Department of Computer ScienceETH Zurich, Zurich, Switzerland; ^2^Department of Psychology, University of PotsdamPotsdam, Germany; ^3^Center for MR-Research, University Children's HospitalZurich, Switzerland; ^4^Children's Research Center, University Children's HospitalZurich, Switzerland; ^5^Department of Child and Adolescent Psychiatry, DRK Kliniken Berlin WestendBerlin, Germany

**Keywords:** learning, intervention, optimization, calculation, spatial representation, interactive learning environment

## Abstract

This article presents the design and a first pilot evaluation of the computer-based training program Calcularis for children with developmental dyscalculia (DD) or difficulties in learning mathematics. The program has been designed according to insights on the typical and atypical development of mathematical abilities. The learning process is supported through multimodal cues, which encode different properties of numbers. To offer optimal learning conditions, a user model completes the program and allows flexible adaptation to a child's individual learning and knowledge profile. Thirty-two children with difficulties in learning mathematics completed the 6–12-weeks computer training. The children played the game for 20 min per day for 5 days a week. The training effects were evaluated using neuropsychological tests. Generally, children benefited significantly from the training regarding number representation and arithmetic operations. Furthermore, children liked to play with the program and reported that the training improved their mathematical abilities.

## Introduction

Arithmetical skills are essential in modern society. However, many children experience difficulties in learning mathematics, ranging from mild to severe numeracy problems. It is therefore important to investigate the typical and atypical development of mathematical abilities as well as intervention approaches to prevent or remediate difficulties. In this study, we present the development of a computer-based training program for children with difficulties in learning mathematics along with case studies and quantitative results of a first evaluation.

In the following, we first introduce different neuro-cognitive models of number processing and numerical development focusing on the models relevant for the design of the training program. We then discuss the potential of computer-based training environments and give an overview of existing interventions before introducing the present study.

### Neuro-cognitive models of number processing and numerical development

Current neuropsychological models postulate distinct representational modules, located in different brain areas, which are relevant for adult cognitive number processing and calculation. One of the first models, the “triple-code model” (Dehaene and Cohen, 1995) comprises a verbal module supporting counting and number fact retrieval, a visual-Arabic module required for solving written arithmetic and an analogue magnitude module (mental number line) for semantic number processing. Lately, an fMRI meta-analysis enabled further insights into supporting and domain-general functions involved in solving arithmetic tasks and suggested a modification and extension of the triple-code model (Arsalidou and Taylor, 2011). Results from functional brain imaging in adults and children indicate that the representation of the mental number line emerges during the first years of school in the parietal lobe due to practice and experiences (Rivera et al., 2005; Ansari and Dhital, 2006; Kucian et al., 2008). The initial assumption of the analogue magnitude representation being notation-independent was challenged in 2007 (Cohen Kadosh et al., 2007). Nieder (2012) recently showed that there are indeed notation-dependent as well as notation-independent neurons responding to numerosity.

While the triple-code model denotes the end state of numerical development, the four-step developmental model (von Aster and Shalev, 2007) describes the path to this end state. It divides the semantic representation (analogue magnitude representation) into an implicit core representation of magnitude and an explicit mental number line, the latter considered as being a “representational redescription” of the former (Karmiloff-Smith, 1992). The (inherited) core-system representation of cardinal magnitude provides the basic meaning of numbers (step 1). Based on this representation, children learn to associate a perceived number with spoken and later written and Arabic symbols. The process of linguistic (step 2) and Arabic (step 3) symbolization is in turn a precondition for the development of a mental number line (step 4). The different representations develop depending on the growing capacity of domain-general functions like working memory.

Lately, other authors have suggested different models of numerical development (Carey, 2001, 2004; Kucian and Kaufmann, 2009; Kaufmann et al., 2011; Noel and Rousselle, 2011; Kaufmann and von Aster, 2012; Vogel and Ansari, 2012). Some authors argue that developmental dyscalculia (DD) is mainly caused by an early, probably genetic, deficit of the basic non-symbolic magnitude system (Butterworth et al., 2011), while others suggest that problems may arise from different developmental reasons, including maladaptive learning experiences and math anxiety (see also the opinion paper Kaufmann et al., submitted). To summarize, there is still an open debate about developmental trajectories and reasons for failure in learning mathematics. However, there seems to be agreement that based on early non-symbolic abilities to access and compare numerical magnitudes, different components of semantic and symbolic representations are developing during childhood and school years. These components develop based on the increasing capacity of domain-general functions and enable a child to successively acquire arithmetic skills.

### Computer-based interventions

The highly complex processes of domain-specific cognitive development need to be taken into account when teaching mathematics. The development of each child's numerical abilities often follows a different speed and is intertwined with the development of other cognitive domains and domain-general abilities (von Aster and Shalev, 2007; Kucian and Kaufmann, 2009; Kaufmann et al., 2011), leading to different mathematical performance profiles (von Aster, 2000; Geary, 2004; Wilson and Dehaene, 2007). Therefore, a high grade of individualization seems necessary.

Educational software can contribute to these requirements. Computer-based trainings can be designed to adapt to an individual child's abilities and provide intensive training in a stimulating environment (Kullik, 2004). The training can for example adapt to cognitive (Naglieri and Johnson, 2000) or to performance profiles of the children (von Aster, 2000; Geary, 2004; Wilson and Dehaene, 2007). This individualization in combination with the fact that the computer is an emotionally neutral medium may also lead to increased motivation and enhance positive self-concepts as every learner gains feelings of success (Ashcraft and Faust, 1994; Spitzer, 2009). Furthermore, computers are an attractive medium for children (Kulik and Kulik, 1991; Schoppek and Tulis, 2010).

In the past years, different meta-analyses have assessed the effects of computer-based instruction, revealing positive results. Kulik and Kulik (1991) and Kulik (1994) computed an average effect size of 0.47 for math learning in elementary school. Other studies reported effect sizes ranging from 0.13 to 0.8 (Khalili and Shashaani, 1994; Fletcher-Flinn and Gravatt, 1995). Li and Ma (2010) found larger effects for elementary school than for higher education and showed that special needs students especially benefit from computer-based instruction.

Existing interventions are, however, mostly conventional. Techniques include training programs for preschool children at risk of developing mathematical difficulties (Griffin et al., 1994; Van De Rijt and Van Luit, 1998; Arnold et al., 2002; Wright, 2003) as well as remedial programs for elementary school children (Van Luit and Naglieri, 1999; Dowker, 2001, 2003; Fuchs et al., 2006; Wilson et al., 2006a; Butterworth et al., 2011; Kucian et al., 2011; Lenhard et al., 2011). Programs designed for preschool children mostly focus on building basic-numerical skills, whereas elementary school trainings target a broader range of skills. Some interventions address basic numerical skills and the establishment of the mental number line (Wilson et al., 2006a), while others train arithmetic fact knowledge (Van Luit and Naglieri, 1999; Fuchs et al., 2006) or are aligned to scholar curricula (Lenhard et al., 2011). Other effective approaches combine the training of basic-numerical capacities with the training of arithmetical knowledge (Dowker, 2001, 2003; Kucian et al., 2011).

The computer-based intervention “Number race” for children with DD trains number comparisons and enhances the links between number and space (Wilson et al., 2006a). Evaluation of the training revealed significant improvements in basic numerical cognition, but the effects did not generalize to counting or arithmetic (Wilson et al., 2006b, 2009; Räsänen et al., 2009). “Rescue Calcularis” is another computer-based intervention for children with DD. It aims to improve the construction and access to the mental number line. The evaluation of the program showed that children with and without DD could benefit from the training (Kucian et al., 2011). “Elfe and Mathis” is a computer-based training (Lenhard et al., 2011) aligned to the German scholar curriculum. Its evaluation demonstrated significant effects. Fuchs et al. (2006) presented a computer-based program to acquire fact knowledge, reporting significant effects in addition. Butterworth et al. (2011) suggest the use of adaptive interactive games for remediation (see also Callaway, 2013). The proposed games train basic-numerical skills (number comparisons and counting) as well as the spatial number representation and simple arithmetic facts. Evaluative results of the training have not yet been published.

The previous studies demonstrate the efficacy of computer-based intervention in number processing. The presented programs however mostly focus on specific skills (such as the training of fact knowledge) and provide only limited adaptability.

### The present study

The objective of the present study is (1) the development of a computer-based training program based on current concepts of numerical development and (2) a first pilot evaluation of its efficacy and practicality. The intervention uses core elements of “Rescue Calcularis” (Kucian et al., 2011). Compared to previous studies, we provide a more complete training of mathematical skills and employ a user model allowing flexible adaptation [based on the student model and control algorithm presented in Käser et al. (2012)]. Our program combines the training of basic numerical cognition with the training of arithmetical abilities. Several past studies (Siegler and Booth, 2004; Booth and Siegler, 2006, 2008; Halberda et al., 2008) have reported significant correlations between math achievement and arithmetical learning and the quality of numerical magnitude representation. In addition, intervention programs that train basic numerical skills and arithmetic knowledge in parallel have proven to be successful (Dowker, 2001, 2003; Kucian et al., 2011). Based on these facts, we expect significant training effects regarding spatial number representation as well as arithmetic performance. Furthermore, we expect an increased motivation by providing an attractive computer-based learning environment and by adapting the difficulty level to the individual child.

## Methods

### Description of the training program

The training program combines the training of basic numerical cognition with the training of different number representations and their interrelations and arithmetical abilities. Our intervention relies on three design principles:

1. Design of numerical stimuli: A special number design enhancing the different number representations is consistently used throughout the training program. Furthermore, the three different number modalities are shown simultaneously at the end of each trial.
2. Adaptability and scaffolding: The learning process of children is different, i.e. they start at different initial levels, progress with different speed and show different specific problems. Our intervention program optimizes the learning process by providing a hierarchically structured learning environment teaching fundamental knowledge first (scaffolding). Furthermore, it is fully adaptive to customize the learning experience of the child. Task difficulty is adapted to the child and specific problems of a child are recognized and addressed.
3. Different types of knowledge: The intervention program aims to balance the acquisition of conceptual knowledge with automation training. Children are taught conceptual knowledge before going over to automation training. An arithmetic operation is for example first introduced and explained. The arithmetic operation and its solution are then modeled using stimuli and finally, mental calculation is trained.

#### Design for numerical stimuli

**Figure 1:**
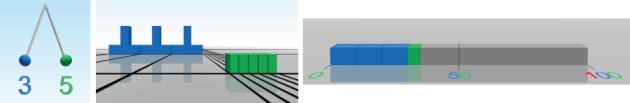
**Numerical stimuli for the number 35.** Number graph (left), colored block (middle) and number line representation with integrated blocks (right).

The special design for numerical stimuli is intended to enhance the different number modalities and to strengthen the links between them. Properties of numbers are encoded with visual cues such as color, form and topology. The digits of a number are attached to the branches of a graph and represented with different colors according to their positions in the place-value system: Units are colored in green, tens in blue and hundreds in red (Figure 1 left). We assume that this representation facilitates the acquisition of the Arabic notation as well as the translation between verbal and Arabic notation. The cardinal magnitude of number is emphasized by representing the number as an assembly of one, ten and hundred blocks. This representation illustrates the fact that numbers are composed of other numbers. The blocks are linearly arranged from left to right (Figure 1 middle) or directly integrated in the number line (Figure 1 right).

#### Structure

**Figure 2:**
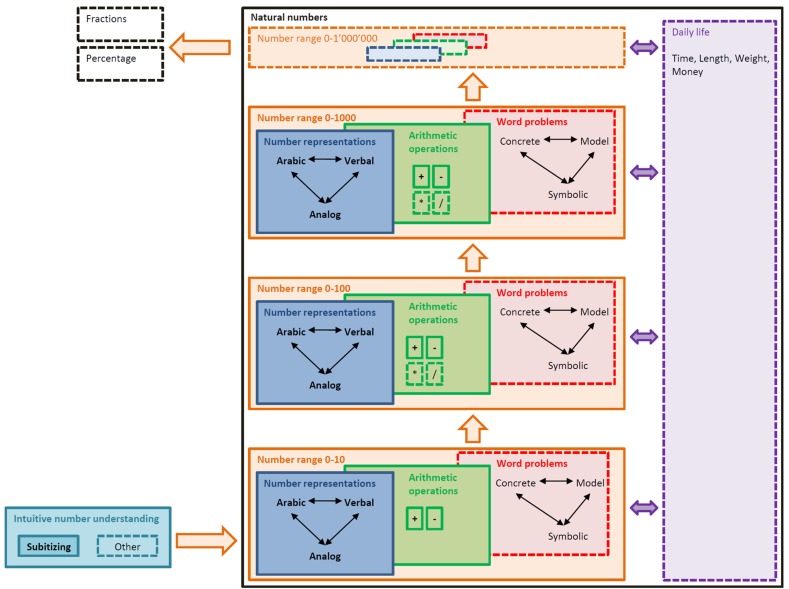
**Target structure of the training program.** The continuous lines mark the areas present in the study version.

The training program is composed of multiple games in a hierarchical structure. Figure 2 shows the target structure of the training program. The study version (section User study) of the program is constrained to specific areas of the target structure (intuitive number understanding, number representations, arithmetic operations) and to natural numbers up to 1000. In the following, we describe the target structure of the training program.

Games are structured along number ranges and further divided into hierarchically ordered areas:

1. Number representations: This area focuses on different number modalities and number understanding in general. It trains transcoding between different number representations. Furthermore, the three interpretations of number are established: Cardinality (quantity), ordinality (position in a sequence) and relativity (difference between two numbers). Games in this area are hierarchically ordered according to the four-step developmental model (von Aster and Shalev, 2007).
2. Arithmetic operations: This area trains arithmetic operations at different difficulty levels. Task difficulty is determined by task complexity, the magnitude of numbers involved and the means (visual aids) available to solve the task.
3. Word problems: A complete understanding of mathematical operations requires the ability to associate a described situation with a mathematical operation and vice-versa. This also presumes an understanding of the actual meaning of the operation.

Each area builds up on knowledge gained in previous areas and implicitly trains these skills further. An additional forth area serves as a precondition for the three areas described above. This area focuses on important precursor abilities (Landerl et al., 2004; Hannula and Lehtinen, 2005; Mazzocco and Thompson, 2005; Krajewski and Schneider, 2009) such as subitizing or counting.

Games can also be categorized based on their complexity and relative importance. Main games are complex games requiring a combination of abilities to solve them. Support games train specific skills and serve as a prerequisite for the main games. Each area features one main game and several support games. A typical training path would traverse each number range from left to right starting with the number range from 0 to 10. The main games are the same for each number range; they just differ by the cardinal magnitude of numbers used.

#### Adaptive algorithm

To offer optimal learning conditions, the training program adapts to the needs of a specific child. All children start the training with the same game. After each trial, the program estimates the actual knowledge state of the child and displays a new task adjusted to this state. The student model and controller mechanism are based on the mathematical concepts presented by Käser et al. (2012) and were developed for the study version of the training structure.

***Student model.*** We model the mathematical knowledge using a dynamic Bayes net. This net consists of a directed acyclic graph representing different mathematical skills and their relationships. The skills are connected based on the dependencies among them, i.e., two abilities A and B have a (directed) connection, if having ability A is a precondition for having ability B. As the skills cannot be observed directly, the program infers them by posing specific tasks and evaluating user actions. Therefore, we assign all types of tasks and their outcome to the different skills. The resulting student model contains 100 different skills. The skills can be assigned to the different areas of the training program.

**Figure 3:**
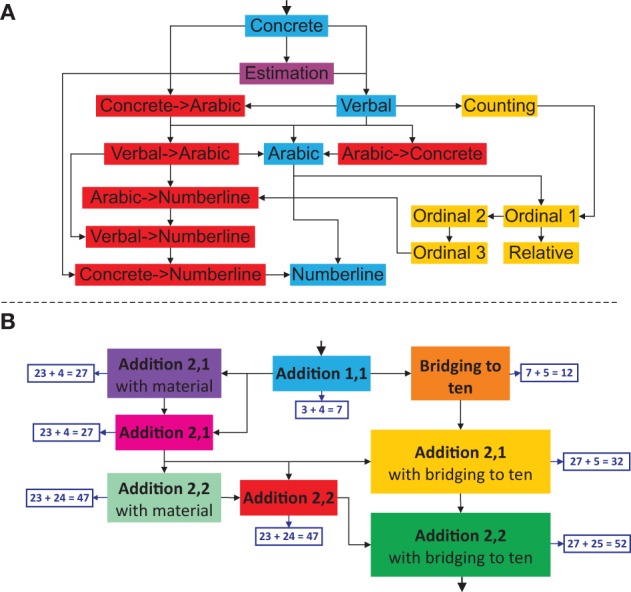
**Extract of the skill net.** Number representation skills from 0 to 100 **(A)**. Addition skill net in the number range 0–100 with example tasks **(B)**.

Figure 3A displays the skills of the area “Number representations” in the number range from 0 to 100. The skills colored in blue denote the different number representations: *Concrete* (number as a set of objects), *Verbal* (spoken number), *Arabic* (written number) and *Numberline* (number as a position on a number line). The transcoding skills (translation between two number representations) are colored in red. The yellow skills introduce the principles of ordinality and relativity of number. Children are required to give the precursor or successor of a number (*Ordinal 1*) or to add/subtract 10 (or 20 or 30) from a given number (*Relative*). In another task, numbers need to be ordered according to their magnitude (*Ordinal 2*). Finally, children need to guess a number in the range from 0 to 100 (*Ordinal 3*). The purple skill trains estimation, i.e., children are required to estimate the quantity of a given point set. Skills in this area are hierarchically ordered according to the four-step developmental model (von Aster and Shalev, 2007). Following the model, the transcoding between the linguistic and Arabic symbolization (*Verbal->Arabic*) is trained before giving the position of a written number on a number line (*Arabic->Numberline*).

The skills in the area “Arithmetic operations” are ordered according to their difficulty. Figure 3B displays the addition skills in the number range from 0 to 100. The difficulty of a task depends on the magnitude of the involved numbers, its complexity and the means allowed solving it. Computing 23 + 24 = 47 (*Addition 2,2*) is considered more difficult than calculating 3 + 4 = 7 (*Addition 1,1*) as the latter task involves smaller numbers. Furthermore, a task involving bridging to 10 such as 27 + 5 = 32 (*Addition 2,1 with bridging to 10*) is rated more complex than a task without any crossing. And finally, modeling the task 23 + 4 = 27 (*Addition 2,1 with material*) is easier than calculating the task mentally (*Addition 2,1*). Subtraction skills in the number range 0 to 100 exhibit exactly the same structure as the addition skills. Therefore, one can obtain the skill net for subtraction by simply replacing *Addition* by *Subtraction* in Figure 3B.

Each skill has two states: A learnt state and an unlearnt state. Having a dynamic Bayes net, the probability for a skill being in the learnt state can be computed. All probabilities are initialized to 0.5, as the system does not know anything about the knowledge of the child. The probabilities are updated after each trial. The probability of a skill can be influenced in different ways. On the one hand, it changes, if the child solves a task that is associated with this skill. On the other hand, solving a task that is associated with a precursor or a successor skill influences the probability.

***Controller.*** The game controller selects the skills for training. After each child input, the controller selects one of the following options based on the probability of the current skill:

1. Stay: Continue the training of the current skill.
2. Go back: Train a precursor skill.
3. Go forward: Train a successor skill.

The decision is based on an upper and lower border for the probability of the current skill. If the probability is larger than the upper border, a more difficult skill is selected for training. If it is smaller than the lower border, a precursor skill is selected for training. The area between the borders is considered as being optimal for training. The two borders have been chosen heuristically to reach the desired behavior: in order to pass a skill, about 10 tasks in a row need to be solved correctly. About five tasks in a row lead to failing a skill.

As a skill can have multiple precursor or successor skills, there are several options for going back or forward. The basic assumption of the model is that in order to pass the current skill, all the precursor skills need to be mastered. If the child therefore fails a skill, the controller selects a precursor skill that has not yet been played for training. When going forward, the control algorithm prefers main games over support games (section Structure) and thus chooses the shortest way through the skill net. A detailed description of the mathematical model and control algorithm can be found in Käser et al. (2012).

**Figure 4:**
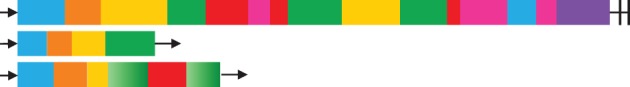
**Skill sequences of three children in addition from 0 to 100.** Colors are consistent with Figure 3B. Users 2 and 3 passed all skills in the range, while user 1 did not pass this range within the training period. The length of the rectangles indicates the number of samples.

At the beginning of the training, children start with the lowest skill in each area. Due to the structure of the skill net and the control algorithm acting on it, each child persecutes a different trajectory through the skill net during training. This variety is increased by repeating less sophisticated skills at random time intervals. Therefore, the path through the network is different for every user (see Figure 4).

To allow an even more accurate adaptation, the program has access to a bug library storing typical error patterns (Gerster, 1982). If a child commits a typical error several times, the controller systematically selects actions for remediation. Table 1 lists the typical error patterns stored in the bug library, along with examples and remediation tasks. For the area of number representations, only one pattern is stored for the landing game: positioning the cone on the wrong side of the indicated center of the number line, i.e., positioning the cone at a number <50 when the given number is >50. For the area of arithmetic operations, a range of error patterns are stored in the bug library. Some of these patterns can be attributed to problems in counting or understanding the basic concepts of addition and subtraction. Remediation skills for these error patterns train simple addition and subtraction tasks with colored blocks (*Addition/Subtraction 1,1 with material*, “slide rule” game). Other error patterns probably occur due to a lack in understanding the Arabic notation system, i.e., the meaning of the different positions of the digits. Selected remediation action for these patterns is the training of the Arabic notation system (*Arabic->Concrete*). Another typical error is the switching of digits (twenty-five is written as “52”) which is remediated by training transcoding from spoken to written numbers (*Verbal->Arabic*). Finally, problems with bridging to 10 are also addressed (*Bridging to ten)*. The bug library was built based on previous work identifying typical error patterns and their causes (Gerster, 1982). In a next step, the typical error patterns will be analysed and refined based on the collected input data.

**Table 1:** **Description of typical errors along with examples and remediation games for the domains of number representations (NR), addition (A) and subtraction (S)**.

	**Description**	**Example**	**Remediation**
NR	Landing game: the child lands the cone on the wrong side of the center (5, 50, or 500).	–	Training of the ordering of numbers according to their magnitude (*Ordinal 1*)
A, S	Correct result is missed by 1 (±1).	5 + 3 = 7	Training of addition or subtraction with colored blocks (*Addition/ Subtraction 1,1 with material*)
A, S	Addition instead of subtraction (or vice versa).	5 + 3 = 2	Training of addition or subtraction with colored blocks (*Addition/ Subtraction 1,1 with material*)
A	Addition of all digits.	12 + 24 = 9	Training of the Arabic notation system (*Arabic->Concrete*)
A, S	Switching of digits when reading/writing a number.	24 − 3 = 12	Transcoding from spoken to written notation (*Verbal->Arabic*)
A, S	Use of wrong digit order.	63 − 5 = 13	Training of the Arabic notation system (*Arabic->Concrete*)
A, S	Forgetting the carry when bridging to ten.	34 + 7 = 31	Training of bridging to 10 using colored blocks (*Bridging to ten*)
A, S	Addition/Subtraction of inner and outer digits.	34 + 13 = 56	Training of the Arabic notation system (*Arabic->Concrete*)
S	Building the difference between digits.	34 − 17 = 23	Training of the Arabic notation system (*Arabic->Concrete*)

#### Games

The training program consists of 10 different types of games that are associated with the presented skills. By varying the numbers used in the games, we obtain 81 different types of tasks (task difficulty levels). In the following, we describe four games of the training program.

**Figure 5:**
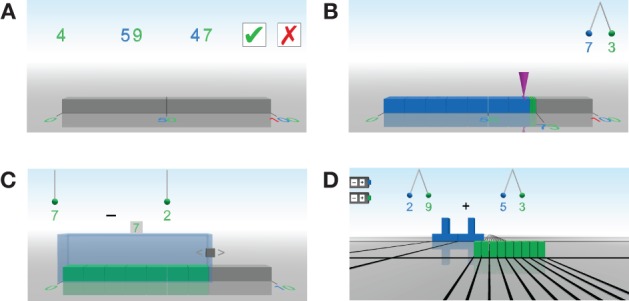
**(A)** “Ordering” game in the range from 0 to 100. **(B)** “Landing” game in the range from 0 to 100. **(C)** “Slide rule” game in the range from 0 to 10. **(D)** Example task of the “Plus and Minus” game.

***Ordering.*** The “ordering” game (Figure 5A) is a support game in the area of “Number Representations,” training ordinal number understanding. A sequence of numbers is displayed for a period of 5 s. Children need to decide, if the sequence was sorted in ascending order. The game is associated with the skill *Ordinal 1* in Figure 3A.

***Landing.*** The “landing” game (Figure 5B) is the main game in the area of “Number Representations,” training spatial number representation. A purple cone must be directed to the position of a given number on a number line (with indicated center), using a joystick. Numbers are presented in verbal or Arabic notation. In another option the cardinality of a given point set and the position of this quantity on the number line have to be estimated. The different modes of the game are associated with the skills *Verbal->Numberline*, *Arabic->Numberline* and *Concrete->Numberline* in Figure 3A. The required accuracy for a correct solution is a deviance of less than 5%.

***Slide rule.*** The “slide rule” game (Figure 5C) is a support game belonging to the area of “Arithmetic operations,” providing an introduction to addition and subtraction using the part-whole concept. An operation task is presented to the child, as well as a number line and a glass case containing a number of unit blocks (according to the first number of the task). The size of the glass case must be changed such that it contains the result of the task. This game would be associated with the skills *Addition 1,1 with material* and *Subtraction 1,1 with material*.

***Plus and Minus.*** The “Plus and Minus” (Figure 5D) game is a support game in the area of “Arithmetic operations.” An arithmetic operation given in Arabic notation must be modeled using colored blocks (one, ten, and hundred). Different strategies are allowed to find the result. This game is associated with all addition and subtraction skills that involve the use of materials.

### User study

#### Study design and participants

The effects of the training program have been assessed in a study with 41 children conducted in Switzerland. Participants were divided into a training group (*n* = 20, 65% females) completing a 12-weeks training and a waiting group (*n* = 21, 66.6% females) starting with a 6-weeks rest period. Comparing the training effects of the training group to those of a waiting group allows controlling for developmental and schooling effects.

Mathematical performance of both groups was evaluated at the beginning of the study (*t*_1_), after 6 weeks (*t*_2_) and after 12 weeks (*t*_3_). Children were required to train with the program 5 times per week, with daily training sessions of 20 min. The groups were matched according to age (training group: *M* = 9.96 years (*SD* = 1.35), min = 7.37, max = 12.06; waiting group: *M* = 9.98 (*SD* = 1.33), min = 7.52, max = 12.21; *t*_(39)_ = −0.04, *p* = 0.96), gender and intelligence (training group CFT-score: *M* = 93.8 (*SD* = 11.9); waiting group CFT-score: *M* = 93.5 (*SD* = 14.1); *t*_(39)_ = 0.07, *p* = 0.95) (Cattell et al., 1997; Weiss, 2006). Groups were built by forming matched pairs of kids, followed by a quasi-random assignment to either the training or waiting group (ensuring that the number of males was balanced between the groups).

All participants were German-speaking and visited the 2nd–5th grade of elementary school. Children were indicated by parents and teachers as exhibiting difficulties in learning mathematic. On average, arithmetic performance [measured with the “Heidelberger Rechentest” HRT (Haffner et al., 2005)] of the participants was around the 10th percentile, corresponding to a T-score of 37 [HRT addition T-score: *M* = 37.15 (*SD* = 7.69); HRT subtraction T-score: *M* = 37.29 (*SD* = 8.77)]. There was no significant difference in arithmetic performance between the groups (HRT addition: *t*_(39)_ = 0.59, *p* = 0.55; HRT subtraction: *t*_(39)_ = −0.63, *p* = 0.53).

Children performed the training at home with exception of one mandatory training session per 6 weeks at our laboratory. Children received a sticker per completed training session that they could put on their training progress sheet. During the training period, all the input data of the children was saved. Therefore, the exact training time of the children could be determined at the end of the study and children with an insufficient number of sessions were excluded from the analysis (see section Results). Parents gave informed consent and children received a small gift for their participation. The presented evaluation was a first pilot study conducted in the context of a large-scale multi-center evaluation study in Germany and Switzerland, which was approved by the ethics committee of the University of Potsdam.

#### Instruments

All children underwent a series of mathematical performance and number processing tests, detailed below. The children completed a questionnaire after the training, including questions on difficulty, motivation, and personal evaluation of the training.

***Heidelberger Rechentest (HRT).*** Arithmetic performance was assessed using the addition and subtraction subtests of the HRT (re-test reliability: addition *r*_*tt*_ = 0.82, subtraction *r*_*tt*_ = 0.86). In these subtests, children are presented a list of addition (subtraction) tasks ordered by difficulty. The goal is to solve as many tasks as possible within 2 min. The maximum number of correct tasks is 40. During the test sessions, the addition subtest of the HRT was always solved first, followed by the subtraction subtests and the computer-based tests described below.

**Figure 6:**
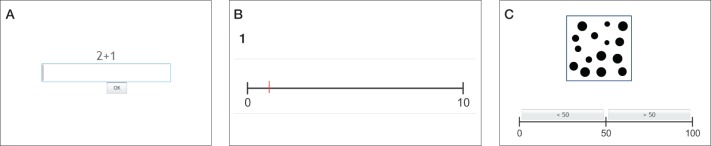
**(A)** Addition task. **(B)** Number line task between 0 and 10. **(C)** Estimation task.

***Computer-based tests.*** Children also underwent a series of computer-based mathematical tests (see Figure 6):

- Arithmetic (AC) (Figure 6A): In this test, children solve a series of addition (subtraction) tasks. Trials are ordered by difficulty and presented serially. The time to solve the tasks is 10 min. The maximum number of solved tasks is 76.
- Number line (NL) (Figure 6B): In this test, children need to indicate the position of a given number (presented in Arabic notation as well as verbally) on a number line. The number line is represented on the screen as a one-dimensional black line with labeled end points. The position of the number can be indicated by mouse-click. There are 10 tasks in the number range from 0 to 10 (NL 10), 20 tasks between 0 and 100 (NL 100) and 10 tasks between 0 and 1000 (NL 1000).
- Non-symbolic magnitude comparison (NC) (Figure 6C): In this test, children are presented 10 sets with 1–9 black dots (excluding 5) for a period of 120 ms. Children need to indicate if the presented number of dots was smaller or larger than 5. The representation of the dots is balanced according to spatial distribution and area properties as described by Rubinsten and Sury (2011). The black area is the same for all trials. Half of the trials have a small extension (high density) while the other half is spread out (low density).
- Estimation: In this task, children are presented twenty sets with 1–99 black dots (excluding 50). Children need to decide, if the presented sets are smaller or larger than 50. Numbers are equally distributed over the range. Confounding visual factors are controlled as described in the non-symbolic magnitude comparison task. Stimuli are shown for a period of 5 s.

During the test sessions, the different tests were solved in the following order: AC addition, NL 0–10, NC, AC subtraction, NL 0–100, estimation, NL 0–1000. The computer-based tests exist in three parallelized versions (one per measurement point). The versions were parallelized according to content and item difficulty. Each version of the addition and subtraction tests for example contains the same number of tasks between 0 and 10 and the same number of tasks involving bridging to 10.

***Feedback questionnaire.*** Children completed a training evaluation questionnaire at the end of the study (*t*_3_). Children indicated for each game, how much they liked it. The scale was represented through smileys, going from a laughing (4) to a crying (0) smiley. The difficulty of the training was judged on a scale from very easy (0) to very difficult (4). And finally, children needed to indicate if the training helped them on a scale from not true (0) to absolutely correct (3).

## Results

Only children with at least 24 sessions after the 6-weeks training period were included in the evaluation of the training. Thus, five children from the training group (4: technical challenges, 1: <24 training sessions) and four children from the waiting group (1: abort of study, 3: <24 training sessions) were excluded from the analysis. The exclusions did not change the matching of the groups. Table 2 gives an overview of the training statistics.

**Table 2:** **Training statistics [Means (*SD*)] of training group (*n* = 15) and waiting group (*n* = 17)**.

	**6-weeks period (*t*_1_–*t*_2_) Training group**	**6-weeks period (*t*_2_–*t*_3_) Waiting group**	**12-weeks period (*t*_1_–*t*_3_) Training group**
Number of training sessions	30.2 (3.2)	32.4 (5.2)	49.2 (2.6)
Number of totally solved tasks	1635.0 (293)	1737.0 (266)	2575.0 (414)
Number of solved tasks per session	54.0 (7.2)	54.0 (5.7)	52.2 (7.0)
Highest reached skill ^a^ (Number representations)	38.6 (8.3)	38.7 (8.4)	40.5 (6.8)
Highest reached skill ^a^ (Arithmetic operations)	40.5 (14.7)	39.1 (15.5)	43.0 (15.1)

a The skills of the adaptive model are divided into the content areas of the training program (section Adaptive algorithm). Skills in each area are ordered by their number, with the easiest skill having the lowest number.

### Quantitative analyses

A repeated measures general linear model (GLM) analysis was conducted to evaluate training effects (*t*_1_ – *t*_2_) as a within-subject factor and group (Training/Waiting) as a between-subject factor. *Post-hoc* paired-sample *t*-tests were used to test for differences in performance for consecutive testing periods (*t*_1_ − *t*_2_, *t*_2_ − *t*_3_). Effect sizes were computed according to Field (2009). No corrections for multiple testing were applied. Table 3 summarizes the means and standard deviations of the behavioral measures for all measurement points, including calculated statistical results. There were no between-group performance differences prior to the intervention.

**Table 3:** **Training effects of training group *TG* (*n* = 15) and waiting group *WG* (*n* = 17) on mathematical performance [Means (*SD*)]**.

**Mathematical performance**		***t*_1_**	***t*_2_**	***t*-score (*t*_2_–*t*_1_)**	***F*-score^d^**	***t*_3_**	***t*-score (*t*_3_–*t*_2_)**	***ES^e^***
AC Addition^a^	*TG*	25.9 (10.8)	30.0 (14.1)	2.38^*^	3.26^+^	34.4 (17.7)	1.95^+^	0.31
	*WG*	27.4 (10.7)	26.1 (12.0)	−0.56		29.4 (11.7)	1.90^+^	
AC Subtraction^a^	*TG*	19.2 (12.7)	24.7 (17.1)	2.77^*^	5.32^*^	28.8 (17.9)	1.99^+^	0.39
	*WG*	19.6 (10.2)	18.9 (10.7)	−0.39		26.3 (14.5)	4.11^**^	
NL10, mean^b^	*TG*	13.2 (10.1)	9.3 (10.6)	−2.06^+^	2.56	7.5 (5.0)	−0.81	0.28
	*WG*	10.3 (10.4)	12.3 (11.6)	0.65		6.1 (4.2)	−2.70^*^	
NL 10, var^c^	*TG*	10.2 (6.3)	6.9 (6.5)	−2.58^*^	4.92^*^	6.7 (4.6)	−0.15	0.38
	*WG*	7.4 (6.4)	9.4 (7.6)	1.02		4.9 (3.3)	−3.10^**^	
NL100, mean^b^	*TG*	10.2 (4.8)	9.6 (6.4)	−0.62	0.98	7.6 (3.3)	−2.24^*^	0.18
	*WG*	13.5 (6.0)	11.3 (5.7)	−1.72		9.3 (7.0)	−1.35	
NL100, var^c^	*TG*	7.7 (3.4)	8.2 (5.1)	0.39	0.47	6.2 (3.0)	−2.15^*^	0.12
	*WG*	9.7 (4.3)	9.1 (5.2)	−0.59		8.2 (5.8)	−0.79	
NL1000, mean^b^	*TG*	18.5 (10.9)	16.1 (7.5)	−1.61	0.70	12.9 (6.7)	−1.79^+^	0.15
	*WG*	18.0 (7.3)	17.4 (8.1)	−0.44		12.6 (5.6)	−4.20^**^	
NL 1000, var^c^	*TG*	13.3 (7.1)	11.9 (5.7)	−0.84	0.00	10.0 (6.4)	−1.01	0.00
	*WG*	13.5 (5.6)	12.0 (5.7)	−1.13		10.0 (4.4)	−1.87^+^	
Estimation^a^	*TG*	15.1 (3.9)	14.9 (3.2)	−0.12	2.85	15.8 (2.1)	1.25	0.29
	*WG*	13.6 (4.5)	16.3 (2.2)	2.25^*^		15.9 (2.9)	−0.61	
NC^a^	*TG*	7.9 (1.9)	8.1 (1.5)	0.39	0.21	8.4 (1.6)	0.59	0.08
	*WG*	7.4 (2.3)	7.8 (2.2)	0.94		8.0 (1.8)	0.19	
HRT Addition^a^	*TG*	18.7 (5.4)	20.4 (5.6)	2.47^*^	0.81	22.5 (5.4)	3.46^**^	0.16
	*WG*	18.5 (4.8)	19.4 (4.3)	1.27		20.4 (5.7)	1.5	
HRT Subtraction^a^	*TG*	15.3 (6.1)	19.8 (5.3)	4.85^***^	11.38^**^	20.2 (6.2)	0.59	0.52
	*WG*	16.9 (6.3)	16.9 (5.6)	0.06		18.4 (5.7)	1.5	

+ *p < 0.1*,

* *p < 0.05*,

** *p < 0.01*,

*** p < 0.001.

a Number of correctly solved tasks.

b Distance (percentage) from correct position.

c Variance of distance (percentage) from correct position.

d Time (t_1_–t_2_) × group.

e Effect sizes of interaction time (t_1_–t_2_) × group. r = 0.10: small effect, r = 0.30: medium effect, r = 0.50: large effect.

#### Arithmetic (AC addition and subtraction)

The interaction between training and group was significant for subtraction (*p* = 0.028) and showed a trend for addition (*p* = 0.081). Both operations demonstrated medium effect sizes (subtraction: *r* = 0.39, addition: *r* = 0.31). The prolongation of the training from 6 to 12 weeks (*t*_2_ − *t*_3_) yielded an additional trend of improvement (addition: *p* = 0.072; subtraction: *p* = 0.066).

#### HRT (addition and subtraction)

The interaction between training and group was significant only for subtraction (subtraction: *p* = 0.002; addition *p* = 0.375), where children showed a large effect size (*r* = 0.52). The prolongation of the training yielded an additional improvement, which was significant only for addition (*p* = 0.004).

#### Number line

The quality of the spatial number representation was measured by calculating the distance (percentage) and the variance of the distance between the correct and the indicated location of the number on the number line. In the number range from 0 to 10, children tended to locate the correct position on the number line more accurately after training (*p* = 0.058) and showed decreased variance (*p* = 0.022). The interaction between training and group was significant only for the variance (mean: *p* = 0.12; variance: *p* = 0.034). Children demonstrated medium effect sizes for both measures (mean: *r* = 0.28, variance: *r* = 0.38). The prolongation of the training did not yield any further benefit. In the number range from 0 to 100, interaction between training and group was not significant (mean: *p* = 0.33; variance: *p* = 0.50). The prolongation of the training had a beneficial effect (mean: *p* = 0.042; variance: *p* = 0.05). In the number range from 0 to 1000, children tended to locate the numbers more accurately only after 12 weeks (mean: *p* = 0.096; variance: *p* = 0.331).

#### NC and estimation

In these two tasks, the interaction between training and group was not significant (estimation: *p* = 0.11; NC: *p* = 0.65). Unexpectedly, the waiting group showed a significant improvement in the estimation task (*p* = 0.039). This significant result stems from outliers with large improvement (children with 2 correct answers at *t*_1_ and 17 correct answers at *t*_2_) due to not understanding the task at *t*_1_.

#### Feedback questionnaire

Children generally liked the training [average over all games: *M* = 3.0 (*SD* = 0.55), scale: 0–4] and rated its difficulty as appropriate [*M* = 1.7 (*SD* = 0.74), scale: 0–4]. They also reported that the training helped them to improve in mathematics [*M* = 2.1 (*SD* = 0.89), scale: 0–3].

### Case studies

To illustrate the concept of the learning program and the operation of the controller, the path through the skill net and the training success of a few children is described in the following. The children and their training characteristics are described in Table 4. The analyses stem from the 6-weeks training period.

**Table 4:** **Characteristics of the three example cases**.

	**Sex**	**Age**	**Class**	**Played sessions**	**Solved tasks**	**Tasks per session**
Anne	Female	8;11	3	28	1272	45.4
Eva	Female	9;8	4	33	1803	54.6
Jane	Female	9;10	4	33	1795	54.4

#### Subtraction 0−100

**Figure 7:**
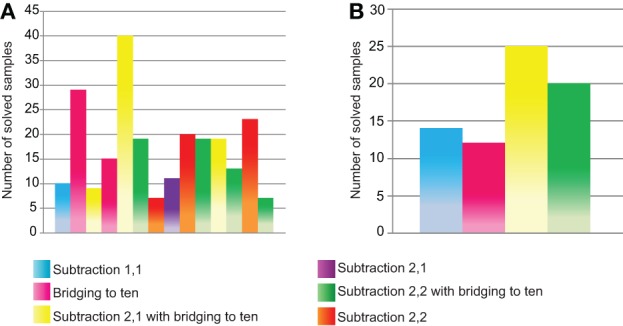
**Number of played samples per skill for Anne (A) and Jane (B)**.

For subtraction in the range from 0 to 100, the course of training (path through the skill net) has been analyzed for Anne and Jane. Figure 7 illustrates the sequence of skills of the two children and the respective numbers of samples.

From Figure 7, it can be seen, that the path through the skill net is different for each child. While Jane took the straight path through the subtraction section, the path of Anne exhibits several branches as she had to go back and consolidate more basic skills. Furthermore, Jane needed in total only 71 samples to pass the subtraction 0–100 section, whereas Anne solved 241 samples to work through the section. The external training effects in subtraction from 0–100 (measured by the AC subtraction test, section Instruments), support this result. In the initial measurement before the training, Jane solved in total 40 tasks, 39 of them correct. She was already proficient in subtraction tasks between 0 and 100 before the training. In contrast, Anne solved in total 26 tasks, 10 of them correct. After the training, Anne managed to solve 23 tasks correctly; she especially improved in subtraction involving bridging to ten. Also Jane showed an improvement after the training, she solved 49 tasks correctly. However, most of her improvement stems from subtraction tasks in the range from 0 to 1000 (the AC subtraction test contains 32 tasks between 0 and 100; the rest of the tasks is in the range from 0 to 1000).

#### Number line 0−100

**Figure 8:**
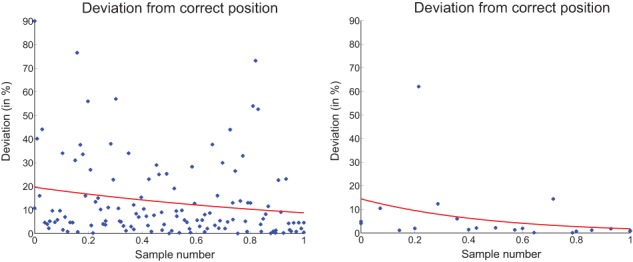
**Landing accuracy over the training from Eva (left) and Jane (right)**.

For Eva and Jane, the ability to place a number on a number line (between 0 and 100) was compared. Before the training, Eva managed the task with an average deviation of 11.4 % (measured by the NL 0–100 test, section Instruments). In contrast, Jane reached an average deviation of 5.4%. Thus, Jane was already more accurate than Eva at the beginning of the training. This fact was confirmed during the course of the training. While Eva needed 127 samples, to pass the landing game (see Figure 5B), Jane passed the game with only 21 samples. The maximum deviation for a sample to be rated as correct was 5%. Figure 8 displays the improvement curves over the course of the training. Recorded input data from all children shows that most samples exhibit an error of 0–20% with only a few samples lying above this range. Therefore, fitting has been done using a generalized linear regression model, assuming a Poisson distribution of the data. The sample indices have been normalized between 0 and 1.

The training sequences of the two children show the same picture. Jane took the direct path through the skill net, whereas Eva had to go back several times. After the training, Eva achieved an average deviation of 6.5% in the NL 0-100 test and Jane's average deviation was 5.1%. Whilst Eva improved significantly, Jane stagnated on a high level.

## Discussion

Although many children experience difficulties in learning mathematics, few studies have investigated targeted interventions based on neuro-cognitive findings of the typical and atypical development of mathematical abilities. Only a fraction of these are computer-based. In the present project, we developed a computer-based intervention targeting children with difficulties in learning mathematics and performed a first evaluation. The results achieved are promising and show significant improvements in subtraction and number representation. Moreover, they confirm the behavioral effects obtained in a previous study employing the computer-based training program “Rescue Calcularis” (Kucian et al., 2011).

### Training

The first pilot study was conducted not only to assess the efficacy of the training program but also the practicality and adaptability of the learning environment. Feedback from children, who have completed the training and rated the difficulty level of the learning program as appropriate, confirms that the quality of the adaptation and the estimation of the children's knowledge were sufficient. Moreover, the need of adaptation to the level of each child is demonstrated by the case studies (section Case studies). As seen in the pre-tests, each child starts with a different amount of mathematical knowledge and shows deficits in different areas. This is also reflected in the course of training: the path through the skill net varies across children. The case studies illustrate that children practice in areas, where they have deficits and generally demonstrate large improvements in these areas after training. Furthermore, it has been shown, that the use of a skill net allowing for different training trajectories optimizes the learning process (Käser et al., 2012).

The evaluation of the feedback questionnaire also supports the improvement of mathematical performance measured in the external tests: On average, children reported that the training had improved their mathematical performance. This subjective feeling of improvement and learning success might also enhance positive self-concepts (Ashcraft and Faust, 1994; Spitzer, 2009). Moreover, children also indicated that they liked to train with the program. The popularity of the learning environment is beneficial as training can only be successful if the children are motivated. Furthermore, the finding demonstrates that the computer is an attractive medium for children and is in line with previous studies (Kulik and Kulik, 1991; Schoppek and Tulis, 2010; Kucian et al., 2011).

### Behavioral effects

Our first results reveal positive training effects in mathematical skills after completion of the training. Children significantly improved their subtraction skills over the course of the 6-weeks-training: They were not only able to solve more complex subtraction problems (medium-large effect in the computer-based subtraction test) but also solved subtraction tasks faster (large effect in HRT). This improvement in subtraction supports the notion of a better mathematical understanding as subtraction is considered as a main indicator for the development of the spatial number line representation (Dehaene, 2011). Furthermore, the decrease in problem solution times can be seen as a shift to increased fact retrieval (Geary et al., 1991; Lemaire and Siegler, 1995; Barrouillet and Fayol, 1998; Jordan et al., 2003). Compared to subtraction, children demonstrated smaller effects in addition (medium effect in computer-based addition test). This may be due to the adaptive nature of the intervention: Addition and subtraction tasks are trained in parallel for each difficulty level. As children performed better in addition at the pre-test, they received more training in subtraction during the intervention. Interestingly, the waiting group did not show significant training effects in the HRT subtests after their 6-weeks training (*t*_2_ − *t*_3_). This fact might stem from the low number of participants or from the adaptability of the training program leading to a different training trajectory for each child.

Children were also able to locate the position of a number on a number line more accurately after training. In the number range between 0 and 10, the deviation from the correct position was reduced by 33% after 6 weeks. Children especially also reduced the variance (medium-large effect size). No further improvement was yield by the prolongation of the training. Yet, most children passed the skills in the number range from 0 to 10 in the first few weeks and thus did not train in this range anymore in the second part of the training. In the number range between 0 and 100, there was no significant interaction. However, the training effect was significant after 3 months (reduction of deviation about 30%). This delay is probably due to the fact, that some of the children arrived at this level only in the second part of the training. Better performance in the number line task indicates refinement of the internal mental number line and more accurate access to it and confirms the results of previous studies (Siegler and Booth, 2004; Booth and Siegler, 2006, 2008; Halberda et al., 2008) which demonstrated significant correlations between arithmetical learning and the quality of numerical magnitude representation.

No significant training effects were observed in the NC and estimation tasks. These results however need to be interpreted with caution because of ceiling effects. At the pre-tests, children solved on average 80% of the NC and 75% of the estimation tasks correctly. Furthermore, some children even reached the maximum score. This result is in line with previous findings (Noel and Rousselle, 2011).

For most of the tasks tested before and after training, prolongation of the training from 6 to 12 weeks yielded a beneficial effect. The improvement of the training group over the whole training period (*t*_1_ − *t*_3_) was significant for all, but the estimation and NC tests. In some tasks (for example NL100 and NL1000), the effects of the second part of the training were similar or higher to those of the first part. This may be due to two facts. Firstly, as the training covers the number range from 0 to 1000, most children had not worked through the whole training after the first 6 weeks. Secondly, the intervention trains different abilities whose effects support each other. However, the supporting effects between those abilities need time to develop (Kaufmann et al., 2003, 2005). The prolongation of the training time from 6 to 12 weeks thus probably led to a strengthening of the mutual effects between the training in number representations and the training in arithmetic operations.

Although a training program focusing on a broad range of mathematical skills and showing a high degree of individualization seems beneficial, it also poses challenges for the evaluation. Firstly, training a variety of skills shortens the training time of each specific skill and thus leads to smaller training effects as mentioned above. Secondly, due to the high adaptability of the program, each child pursues a different training trajectory, i.e., the children train different skills and might even not train a specific skill at all because they either already possessed that ability prior to the training or did not arrive at this difficulty level during training. Therefore, training progress is hard to compare and inconsistencies in training effects may be observed. Nevertheless, the first obtained pilot results are promising and form the basis for further evaluation.

Another important point is the connection to practice. When used in school settings, computer-based training programs might not show significant improvement compared to conventional classroom teaching (Dynarski et al., 2007). However, we believe that carefully designed computer-based training programs provide a valuable addition to conventional classroom teaching in providing a possibility to differentially address individual characteristics.

### Limitations

Some limitations regarding the participants and the study design have to be considered. Firstly, there were no measurements done after a 12-weeks rest period. Thus, for the 12-weeks training period, the training effects could not be compared to the effects of a rest period. Regarding the significant effects of the 6-weeks training, we conclude that also the effects of the 12-weeks training period can be plausibly attributed to the training.

Secondly, children were not tested according to common criteria of DD. Children were indicated by parents and teachers as exhibiting difficulties in learning mathematics. Generally, participants indeed demonstrated a mathematical performance below the 25th percentile in the pre-tests (the four children performing above the 25th percentile had insufficient grades in math). However, as described in section Study design and participants, the participants' mean score even demonstrated an arithmetic performance around the 10th percentile. A further study restricted to children diagnosed with DD is currently conducted in Germany. Nevertheless, our less strict criterion for deficits in mathematical performance seems also informative. It has been shown that the cognitive characteristics of low performing children are indeed dependent on the cut-off criterion used. However, children fulfilling a softer criterion exhibit similar difficulties to those fulfilling stronger criteria, but to a smaller extent (Murphy et al., 2007).

Thirdly, the effects of the training period were only compared to those of a rest period. No comparison to a control training was conducted. As this first pilot study was designed to evaluate the concepts used in the training program and to assess its adaptability, the design used seems sufficient. Having proved the effects of the training in this first step, the mentioned further study conducted in Germany will compare the effects also to a control training.

### Outlook

The presented results from the first evaluation form the basis for further evaluation and improvement of the training program. In a first step, the program is evaluated in a large-scale study conducted in Germany. This study compares effects to a control training and also assesses domain-unspecific measures such as attention, overcoming the limitations of the pilot study.

In a second step, the training program will be improved and extended.

Evaluation of input data and observations of supervised training sessions have shown that children advance too fast in the area of arithmetic operations. Therefore, an incorporation of answer times into the mathematical model is planned, allowing to set time limits for tasks and giving an indication of strategies used (for example fact retrieval versus counting). Furthermore, games training different calculation strategies would be beneficial and put more emphasis on conceptual knowledge (instead of fact knowledge).

The current concept of the training program balances the training time between the area of arithmetic operations and number representations. However, while the area of arithmetic operations trains only addition and subtraction skills, a variety of skills are trained in the area of number representations. Due to this high number of skills, some skills are only trained for a short amount of time and thus no significant improvement in these skills can be observed. The external tests do for example not show any significant improvement in estimation or non-symbolic magnitude comparison tasks. Allocating more training time to the number representations area might solve this issue.

At the moment, the training program entirely relies on intrinsic motivation. Although children indicated that they liked to train with the program, the training could benefit from additional motivational instruments such as the collection of points (for example for correct tasks or training time) and the visualization of learning progress. A version including additional instruments is already planned and will be evaluated in a further user study.

## Conclusion

In the present study, the computer-based training program Calcularis for children with mathematical learning problems was developed and evaluated. The design of the program is based on current neuropsychological findings. The program features a control algorithm allowing adaptation to the user and thus optimization of learning processes. Evaluation of the learning program showed significant training effects in number representation as well as in subtraction. The program proved to adapt well to the needs of the children and feedback from participants was positive. The results obtained from the first evaluation form a promising basis for further evaluation.

### Conflict of interest statement

The authors declare that the research was conducted in the absence of any commercial or financial relationships that could be construed as a potential conflict of interest.
